# Supplementary material for: Patterns of Intron Gain and Loss in Fungi
Source: PLoS Biol. 2004 Nov 30;2(12):e422. doi: 10.1371/journal.pbio.0020422 (PMC532390; doi:10.1371/journal.pbio.0020422)
Supplement: Table S1 — Also available at http://genes.mit.edu/NielsenEtAl/. (4.3 MB ZIP). [file pbio.0020422.st001.zip › NielsenEtAl/html/158.html]

AN8039.1.NCU05347.1.MG06213.1.FG01627.1


```
 CLUSTAL W (1.82) Multiple Sequence Alignments - Introns Inserted


Sequence 1: NCU05347.1	143 aa
Sequence 2: MG06213.1	142 aa
Sequence 3: FG01627.1	144 aa
Sequence 4: AN8039.1	136 aa
Alignment Length: 144 aa
Number Identitical Residues: 117 aa
Alignment Score (without introns) 4376


MG06213.1 	MAGGKGKSSGGKSSGGKTSG-EGPKK-QQSHSARAGLQ0FPCGRVKRFLKQNTQNKMRVG
NCU05347.1	MAGGKGKSSGGKSSGGKTSGVEGPKK-QQSHSARAGLQ0FPCGRVKRFLKQNTQNKMRVG
FG01627.1 	MPGGKGKSSGGKSSGGKTSGTEGANKKQQSHSARAGLQ0FPCGRVKRFLKQNTQQKMRVG
AN8039.1  	MPGGKGKSIGGKAGSKDSAG-----KAQKSHSAKAGLQ0FPCGRVKRFLKNNTQNKMRVG
          	*.****** ***:.. .::*     * *:****:**** ***********:***:*****

MG06213.1 	AKA~AVYVTAVLEYLTAEVLELAG0NAAKDLKVKRITPRHLQLAIRGDEELDTLIRATIA
NCU05347.1	AKA~AVYVTAVLEYLTAEVLELAG0NAAKDLKVKRITPRHLQLAIRGDEELDTLIRATIA
FG01627.1 	AKA~AVYVTAVLEYLTAEVLELAG0NAAKDLKVKRITPRHLQLAIRGDEELDTLIRATIA
AN8039.1  	AKA1AVYVTAVLEYLTAEVLELAG~NAAKDLKVKRITPRHLQLAIRGDEELDTLIRATIA
          	*** ******************** ***********************************

MG06213.1 	FGGVLPHINRALLLKVEQKKKNKQIEA
NCU05347.1	FGGVLPHINRALLLKVEQKKKAKAQEA
FG01627.1 	YGGVLPHINRALLLKVEQKKKAKALEG
AN8039.1  	FGGVLPRINRALLLKVEQKKKKDA---
          	:*****:************** .
```
